# High-throughput X-ray spectroscopy: characterization of the ARDESIA detector with high-end digital pulse processors

**DOI:** 10.1107/S1600577525007738

**Published:** 2025-09-22

**Authors:** Beatrice Pedretti, Giacomo Ticchi, Pietro Barcella, Giacomo Borghi, Marco Carminati, Carlo Fiorini

**Affiliations:** a Dipartimento di Elettronica, Informazione e Bioingegneria Politecnico di Milano Italy; b Istituto Nazionale di Fisica Nucleare (INFN) Sezione di Milano Italy; Bhabha Atomic Research Centre, India

**Keywords:** digital pulse processor, silicon drift detectors, X-ray fluorescence spectroscopy, synchrotron radiation

## Abstract

Among the many factors that determine data acquisition efficiency in synchrotron radiation experiments, detector throughput is a primary bottleneck. As modern synchrotron sources provide increasingly intense X-ray beams, the signal processing time of individual detector channels often becomes the limiting factor for throughput. State-of-the-art digital pulse processors (DPPs) can mitigate this bottleneck by minimizing the processing time per channel, thereby improving overall event throughput. In this work, we evaluate the performance of the ARDESIA-16 detection system, based on a 16-channel monolithic silicon drift detector array, when coupled with two advanced DPPs – DANTE+ and FalconX – which employ different signal processing architectures. Using a standard ^55^Fe source, we systematically characterize and compare output count rate, energy resolution, dead time and spectral quality across a broad range of input count rates, from 40 kcps to 2.7 Mcps. Our results demonstrate the excellent performance of ARDESIA-16 with both DPP systems while highlighting a key trade-off between energy resolution and event throughput that requires thorough optimization for high-rate synchrotron applications.

## Abstract 2

Digital pulse processors (DPPs) have revolutionized X-ray spectroscopy through their advanced real-time signal processing and analysis capabilities. This paper presents a detailed evaluation of the performance of an ARDESIA-16 silicon-drift-detector-based fluorescence detector with two state-of-the-art DPP systems, DANTE+ and FalconX, examining their performance in high-throughput X-ray spectroscopy applications.

## 1. Introduction

X-ray fluorescence spectroscopy (XRF) is a powerful tool for investigating material properties by analyzing their fluorescence emission spectra, supporting researchers in a wide range of applications, including biology, materials science and cultural heritage conservation (Nakano, 2023 ▸; Fitton, 2014 ▸; Revenko & Pashkova, 2023 ▸; Tsuji *et al.*, 2012 ▸; Janssens *et al.*, 2000 ▸). Synchrotron radiation facilities provide high-intensity X-ray sources that enable XRF analysis of trace elements (Gilfrich, 1988 ▸), time-resolved experiments (Monteiro *et al.*, 2021 ▸; Blamey *et al.*, 2018 ▸; Ressler *et al.*, 2004 ▸; Milne *et al.*, 2014 ▸) and spatially resolved mapping at the micro and nanoscale (Ryan *et al.*, 2013 ▸; Lombi *et al.*, 2011 ▸). However, this brilliance presents significant challenges for detection systems, which must handle high photon flux avoiding saturation of the readout electronics while maintaining adequate energy resolution. Moreover, since access to synchrotron facilities is limited by time constraints, efficient data collection is crucial for researchers working with these high-demand resources. Therefore, a high count rate capability of the detection system is critical for exploiting the full potential of modern synchrotron facilities and represents a key parameter in synchrotron radiation experiments, as it directly affects data acquisition efficiency and has driven the development of high-data-throughput energy-dispersive XRF spectrometers (Scholze *et al.*, 2006 ▸; Guerra *et al.*, 2012 ▸; Longoni *et al.*, 2006 ▸).

Two strategies have emerged to enhance event throughput, meaning the rate of successfully processed X-ray photon interactions, in synchrotron radiation experiments: increasing the number of detection channels and using advanced digital pulse processors (DPPs) to maximize per-channel event throughput.

The first strategy involves dividing the detector’s active area into multiple independent channels, each collecting incoming photons, effectively decreasing the photon flux density per unit area. While most multichannel detection systems combine single-element silicon drift detectors (SDDs) or high-purity germanium (HPGe) detectors, this approach creates large dead areas between elements. As a result, modern spectrometer development increasingly favors monolithic configurations. By integrating multiple pixels onto a single silicon substrate, these designs can minimize the dead area between channels, which is a significant advantage over arrays assembled from multiple, discrete single-element detectors. While the solid angle coverage is ultimately determined by the specific geometry and housing of the detector system, the compact nature of monolithic arrays facilitates designs with high geometric efficiency (Fiorini *et al.*, 2002 ▸; Alberti *et al.*, 2006 ▸; Toscano *et al.*, 2024 ▸; Strüder *et al.*, 1998 ▸; Bertuccio *et al.*, 2016 ▸; Lechner *et al.*, 2004 ▸; Gauthier *et al.*, 1996 ▸; Lechner *et al.*, 2001 ▸; Carminati *et al.*, 2023 ▸; Siegmann *et al.*, 2024 ▸). By increasing the number of independent readout channels, the system can process a higher total photon flux without saturating, as each channel handles only a fraction of the incoming events, ensuring that the count rate per channel is kept below the electronics’ saturation level. This allows the beam intensity to be raised, improving the overall output count rate (OCR) through parallel acquisition and signal processing. However, while this approach offers substantial benefits in terms of event throughput optimization, it comes with several technical challenges:

(i) Complexity and cost: more channels require increasingly complex electronics, including advanced signal processing circuits and multiple analog-to-digital converters (ADCs), increasing implementation and maintenance complexity and cost.

(ii) Heat dissipation: additional charge-sensitive amplifiers (CSAs) generate more heat during operation, requiring advanced thermal management solutions to minimize thermal noise and detector leakage current.

(iii) Signal crosstalk: in densely packed systems, readout electronics are particularly susceptible to electromagnetic interference and signal crosstalk, which can potentially degrade spectral quality. Careful design considerations are thus required to maintain signal integrity.

Despite these challenges, monolithic SDD-based designs have proven effective for enhancing event throughput in high-rate synchrotron applications. The ARDESIA project (Section 2) exemplifies this approach by developing high-resolution X-ray spectrometers with high-count-rate capability for synchrotron applications (Utica *et al.*, 2019 ▸; Hafizh *et al.*, 2019*a* ▸; Hafizh *et al.*, 2018 ▸). These spectrometers enable XRF, X-ray absorption spectroscopy and X-ray nanoprobing imaging across an energy range of 0.2–20 keV. Both the ARDESIA-16 spectrometer (Utica *et al.*, 2021 ▸) and the upcoming ARDESIA-64 detector (Pedretti *et al.*, 2024 ▸) offer scalable solutions that address the aforementioned challenges while maintaining high performance.

The second strategy to increase event throughput involves the use of state-of-the-art DPPs to process the analog signals from the X-ray detector’s conditioning stage. DPPs digitize the analog pulses from the charge preamplifier and manage signal elaboration steps such as trigger detection, pile-up rejection (PUR), baseline recovery and peak identification (Goulding, 1972 ▸; Sampietro *et al.*, 1995 ▸; Jordanov *et al.*, 1994 ▸). Compared with traditional analog shaping amplifiers, DPPs offer inherent advantages for applications requiring low noise and high count rates, such as the absence of dead time during peak detection and digitization, resulting in significantly higher event throughput (Abbene *et al.*, 2013 ▸; Hafizh *et al.*, 2019*b* ▸). This means that a DPP can handle a larger volume of pulses within a given time frame. Moreover, the finite impulse response of a digital processor contributes to the reduction of pile-up and other effects associated with pulse overlap, making them especially advantageous at high count rates, where analog systems may encounter limitations.

To address the growing need for ultra-high-rate detectors in next-generation synchrotron beamlines, this study evaluates the performance of an ARDESIA-16 detection module in high-rate measurements using two different state-of-the-art DPPs – DANTE+ (XGLab, 2024 ▸) and FalconX (XIA LLC, 2024 ▸). Performance assessments were conducted under repeatable and controlled measurement conditions, with fixed input count rates (ICRs) and identical operating parameters, isolating the DPP as the only variable. We examine key metrics, including OCR, dead time, spectral quality and energy resolution, measured as the full width at half-maximum (FWHM) of the 5.9 keV Mn-*K*_α_ peak of a standard ^55^Fe source. These parameters are essential indicators for assessing the performance of energy-dispersive XRF spectrometers in high-rate environments. Our measurements were specifically optimized to achieve a reasonable compromise between energy resolution and a high count rate with low dead time. While a full characterization across a wider energy range would provide interesting insight into the system’s capabilities, the use of a ^55^Fe source, providing a monoenergetic peak at 5.9 keV, offers a standard benchmark for evaluating the core performance of the signal processing chain.

In Section 2 we detail the experimental setup and provide essential specifications for the different DPPs. In Section 3 we present the results of our performance evaluations, and we discuss the implications of our findings for future research and applications.

## 2. Experimental setup and methodology

**Figure 1:**
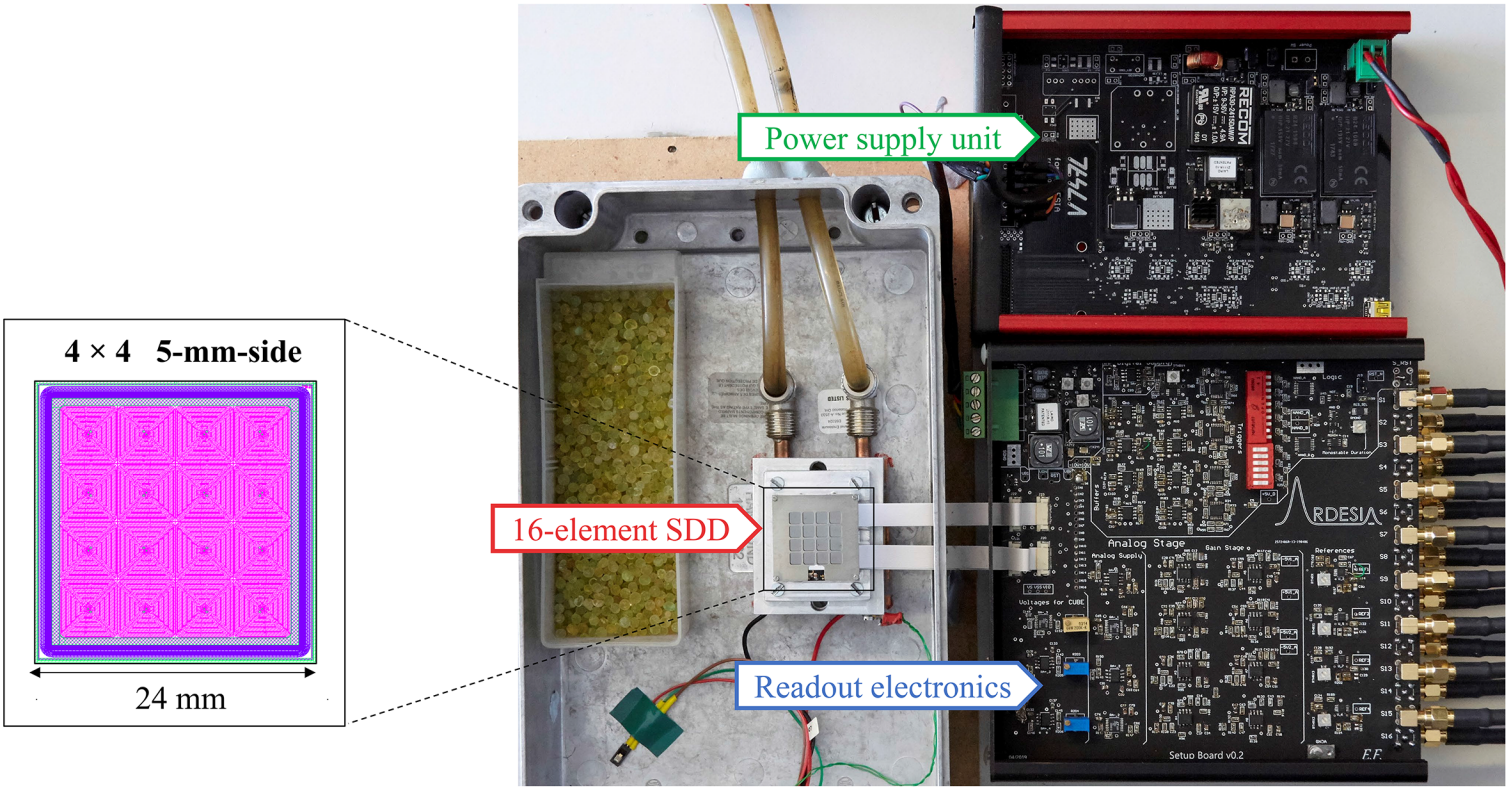
Characterization setup of the detection module highlighting key components, such as the 16-channel SDD module, readout electronics and biasing electronics. On the left, the layout of an ARDESIA-16 SDD.

The performance evaluation utilized a detection module based on a collimated 16-channel monolithic SDD array manufactured by Fondazione Bruno Kessler (Trento, Italy). This detector featured a 450 µm-thick active region within a 24 mm × 24 mm die area, with each of the 16 channels configured in a square geometry measuring 5 mm per side (Fig. 1 ▸). A 500 µm-thick molybdenum collimator grid was mounted in front of the detector surface to minimize charge sharing between adjacent channels. The collimator grid, aligned with the 4 × 4 channel array, introduces a geometric dead area of 19%. Signal acquisition was performed through four custom-designed four-channel pulsed-reset CUBE CSAs (Bombelli *et al.*, 2011 ▸; Quaglia *et al.*, 2015 ▸) specifically optimized for low-noise performance in X-ray spectroscopy applications.

The detection module was enclosed in a light-tight aluminium housing to shield against ambient light interference. Inside the enclosure, relative humidity was maintained below 5% using silica gel desiccants, preventing moisture accumulation on both the detector surface and carrier printed circuit board. Temperature control was achieved using a two-stage thermoelectric cooler coupled to a liquid-cooled heat sink, maintaining the detector at an operating temperature of −33.4 ± 0.7°C.

A custom-designed biasing unit (Hafizh *et al.*, 2019*c* ▸) was used to provide power supply to the setup electronics, which was designed to manage signal conditioning of the CSA outputs, deliver a programmable threshold-sensitive reset signal to all preamplifiers, and provide ultra-low-noise reference and power supply to the CSAs and the SDD array.

To systematically evaluate detector performance across a wide range of ICRs, we integrated a custom 3D-printed source holder with adjustable height, allowing controlled modulation of an uncollimated ^55^Fe source’s distance from the detector surface. This configuration allowed precise modulation of the source-to-detector distance, enabling measurements spanning from 40 kcps ICR to 2.7 Mcps ICR using a single radioactive source. For each point in the comparative analysis, the radioactive source was set to a fixed position, and the detector signal was then processed by each DPP in turn. This procedure ensured that the ICR was identical for both systems under any given test condition, isolating the DPP as the only variable.

While this configuration limited analysis to a single detector channel, the performance uniformity of the 16-channel array had been established in a prior characterization. Those tests, conducted at low count rate, demonstrated excellent consistency across all 16 channels. Based on this confirmed uniformity, a single channel was selected as representative, providing consistent and reproducible experimental conditions, essential for evaluating detector performance when paired with different DPPs and operated across varying count rate conditions.

### 2.1. Digital pulse processor systems

The evaluation of ARDESIA-16’s maximum throughput capabilities involved testing with two state-of-the-art DPP systems that implement fundamentally different signal processing approaches. This comparative analysis was designed to assess ARDESIA-16’s versatility and robustness when integrated into different beamline configurations.

The first processor, DANTE+ (XGLab, 2024 ▸), implements trapezoidal filtering algorithms for pulse height analysis. This approach, based on established digital signal processing techniques (Jordanov *et al.*, 1994 ▸; Jordanov & Knoll, 1994 ▸), processes charge pulses using a trapezoidal weighting function, ensuring precise energy determination.

The second system, FalconX (XIA LLC, 2024 ▸), employs model-based pulse processing algorithms (Scoullar *et al.*, 2011 ▸), utilizing mathematical modeling of pulse shapes to extract energy information and dynamically adapting to measured pulse shapes. This method is designed to improve pulse deconvolution and pile-up handling in high-rate measurement conditions.

Both systems were configured to interface with the ARDESIA-16 detection module using standardized connections, enabling a direct comparison of their respective performance characteristics when processing identical detector signals under controlled experimental conditions.

#### 2.1.1. DANTE+ DPP

**Figure 2:**
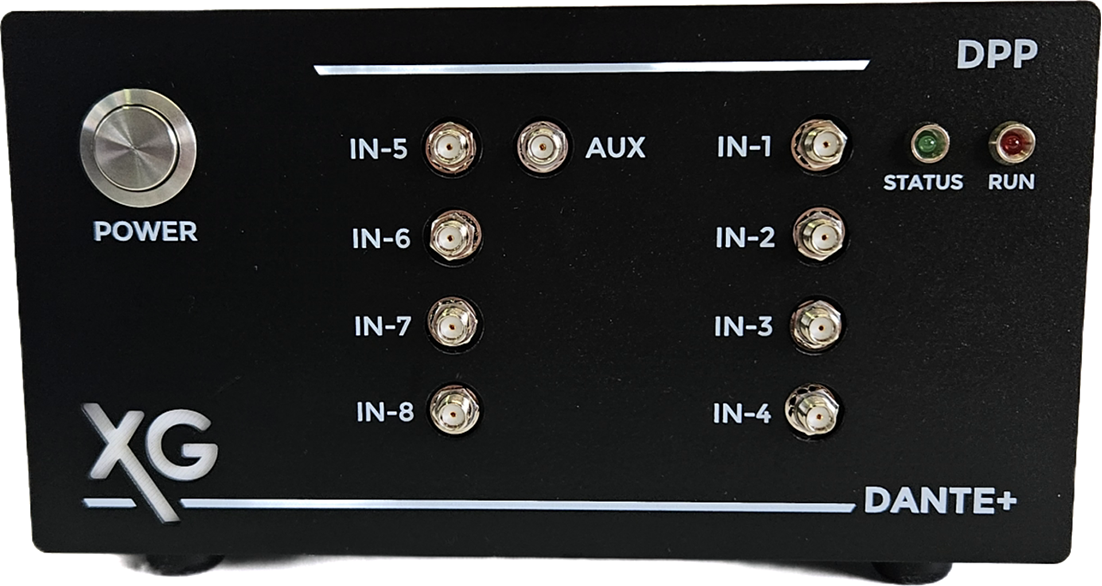
The DANTE+ DPP.

DANTE+ (Fig. 2 ▸), presented by Bombelli (2023 ▸), is the latest X-ray spectroscopy DPP developed by XGLab srl (Milano, Italy) and is based on the architecture of its predecessor, DANTE, which was extensively evaluated and characterized by Iguaz *et al.* (2023 ▸).

The frontend of DANTE+ includes a 16-bit ADC operating at 125 MHz for high-resolution signal digitization. The hardware supports both AC and DC coupling modes; for our measurements, we used DC coupling as it proved more suitable for the intended application. Multiple configurations are possible (1, 4 and 8 channels per enclosure) with a daisy-chain design extendable to 32 channels while maintaining synchronized timing across channels, making it suitable for large-scale experimental setups.

The signal processing chain is based on an implementation of trapezoidal filtering (Jordanov *et al.*, 1994 ▸), which models the signal from the charge-sensitive amplifier (CSA) with a flat-topped trapezoid. This implementation supports both pulsed reset and continuous reset strategies, making it suitable for low-energy X-ray detection and capable of processing signals down to the beryllium fluorescence line. The primary advantage of this approach is its ability to minimize the effects of electronic noise while maximizing energy resolution. The system’s implementation of trapezoidal filtering allows for control over the shaping parameters, allowing adjustments to both peaking time and flat-top duration in 8 ns increments. The signal processing chain also incorporates baseline restoration algorithms that monitor and correct for baseline shifts, ensuring measurement stability even under high count rate conditions.

DANTE+ supports multiple data acquisition modes tailored to different experimental requirements. The system implements single-spectrum acquisition, parameter sweep, list and list-map and mapping modes, with enhanced trigger/gate synchronization capabilities in mapping mode.

**Figure 3:**
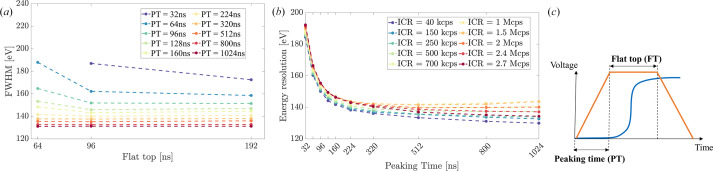
Trapezoidal parameters tuning performed with DANTE+ DPP. (*a*) Energy resolution (quoted as FWHM of the Mn-*K*_α_ peak) as a function of the trapezoidal filter’s flat top (FT) for varying values of peaking time (PT) parameter. (*b*) Energy resolution as a function of the trapezoidal filter’s PT for varying ICR. (*c*) Schematic representation of trapezoidal filter parameters.

Initial optimization focused on establishing optimal operating parameters for each system. For DANTE+, this involved systematic evaluation of trapezoidal filter parameters, depicted in Fig. 3 ▸(*c*). The flat-top time was optimized first [Fig. 3 ▸(*a*)], with 96 ns identified as providing optimal performance for balancing ballistic deficit prevention (Loo *et al.*, 1988 ▸) with high count rate capabilities. This parameter was then fixed for subsequent measurements to ensure consistent experimental conditions.

As for peaking time (PT) optimization, DANTE+ demonstrated comparable performance across a broad range of ICRs. Fig. 3 ▸(*b*) represents the energy resolution as a function of the trapezoidal filter PT, for varying ICRs. It can be observed that energy resolution is below 190 eV for all the tested ICRs even with short PT values, and consistently improves with increasing PT up to 1 µs, dropping below 140 eV for all tested ICRs, with a minimum value of 125 eV achieved at the lowest ICR for a PT of 2 µs.

FalconX, with its model-based approach detailed in Section 2.1.2, required different optimization strategies focused on filter selection rather than traditional shaping parameters.

#### 2.1.2. FalconX DPP

**Figure 4:**
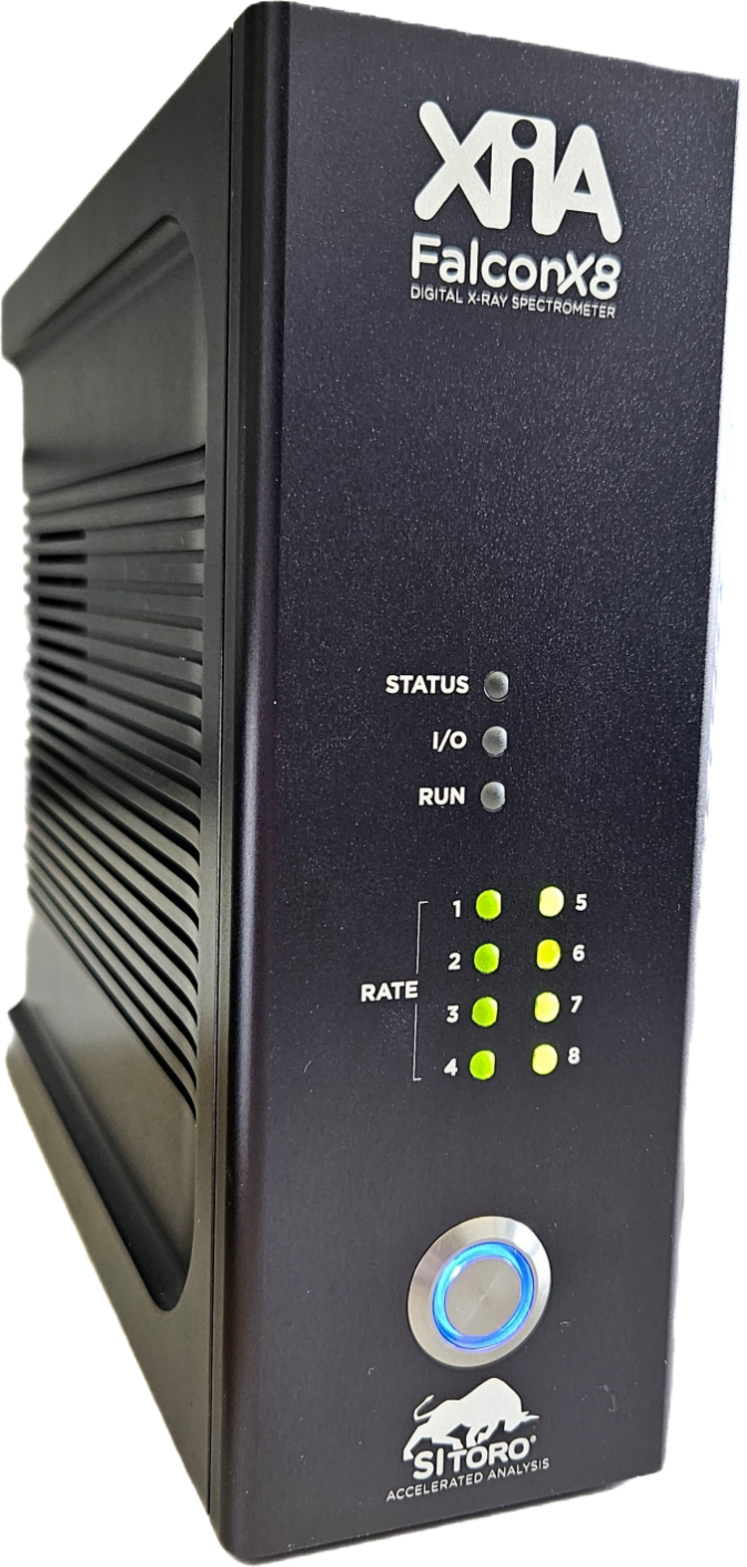
The FalconX DPP.

The FalconX DPP (Fig. 4 ▸) (XIA LLC, 2024 ▸) has been developed for high-throughput X-ray spectroscopy and is designed to support experiments requiring high count rates with accurate energy resolution. The system here used is configured with eight parallel channels and is designed with an expandable rack-mount architecture, allowing its integration into both small-scale laboratory setups and large-scale synchrotron facilities. Each channel features a 16-bit ADC operating at 250 MHz, which ensures accurate digitization of detector signals while accommodating both DC and AC coupling to interface with different detector outputs. Our measurements were best performed with AC coupling modality. Fast mapping and list mode acquisition options allow for time-resolved event recording, and the system achieves a timing resolution of less than 1 ns.

A central feature of the FalconX DPP is its implementation of the proprietary SITORO algorithm (Southern Innovation, 2025 ▸), which employs a model-based processing approach. Unlike trapezoidal filtering techniques that rely on fixed shaping parameters, the SITORO algorithm utilizes the unit impulse response derived from the detector characterization stage. This response is constructed by averaging a large number of individual radiation events, allowing the algorithm to account for the expected pulse shape from the detector (Scoullar *et al.*, 2011 ▸), and incoming pulses are compared in real time against these models. This approach offers inherent advantages in pile-up handling, as rather than simply rejecting overlapping pulses the system can deconvolve them to extract energy information from the individual events. This is particularly advantageous under high-flux conditions and contributes to the system’s good performance at high ICR (Bordessoule *et al.*, 2019 ▸).

In addition to its pulse recovery capabilities, the FalconX DPP incorporates advanced baseline restoration techniques that continuously monitor the baseline level and apply dynamic corrections to counteract any drift during prolonged high-rate operation. This functionality is important for maintaining the stability of energy measurements, as baseline shifts can adversely affect the FWHM of significant spectral peaks.

## 3. Experimental results and discussion

Parameter optimization was first performed at low count rates (approximately 40 kcps) to establish baseline performance for both systems. For DANTE+, this involved systematically optimizing flat-top and peaking time parameters (Section 2.1.1). The optimal flat-top duration was determined to be 96 ns, which maximized energy resolution while reducing ballistic deficit effects. This flat-top value remained constant throughout all measurements.

For the FalconX, to create a stable basis for comparison, hardware parameters were fixed to previously optimized values found during earlier synchrotron measurements with the ARDESIA detector. The optimization performed for this study was therefore focused exclusively on empirically selecting the processing filter that provided the best trade-off between energy resolution and event throughput. The high rate (HR) filter was ultimately determined to be optimal for the ARDESIA detector configuration.

Following the parameter optimization, several key performance metrics were evaluated, including energy resolution, count rate capability and peak stability. The measurements were conducted under optimized conditions for each system, with careful attention to maintaining comparable parameters and operating conditions for both systems to ensure a fair analysis.

### 3.1. Energy resolution

The energy resolution was measured as the FWHM of the Mn-*K*_α_ peak at 5.9 keV of the ^55^Fe source. To ensure consistent analysis, an identical double Gaussian fitting algorithm was implemented to calibrate the *K*_α_ and *K*_β_ peaks and characterize the energy resolution across both systems.

**Figure 5:**
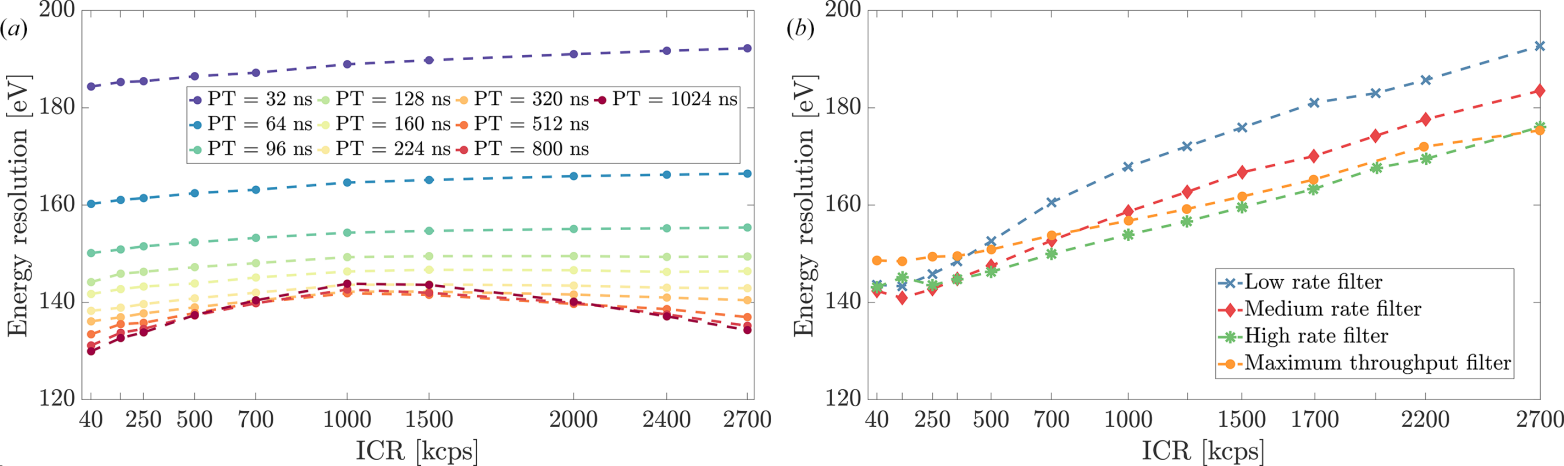
Energy resolution dependence on ICR measured respectively with (*a*) DANTE+ and (*b*) FalconX. The gradual resolution degradation highlights the challenges of maintaining high-quality spectroscopic measurements under increasing experimental demands. (*a*) Multiple lines represent different peaking time values, stressing the importance of optimizing the shaping time parameters for accurate spectral performance. (*b*) The different lines represent the detection system’s behavior across various FalconX processing filters.

Energy resolution measurements revealed distinct performance characteristics for each system. When specifically configured for optimal energy resolution at low count rates, around 40 kcps, ARDESIA achieved its best resolution performance of 125 eV FWHM for the Mn-*K*_α_ peak when paired to DANTE+, and 135 eV when paired to FalconX. However, for the systematic characterization presented in this work, a trade-off between optimal energy resolution and high event throughput was necessary. Consequently, a single, fixed set of parameters optimized for high-rate performance was used for all subsequent measurements. With these settings, the best energy resolution at low ICR was approximately 131 eV for DANTE+ and 140 eV for FalconX. As ICR increased, both systems showed interesting behavior in their energy resolution response (Fig. 5 ▸). DANTE+, with its trapezoidal filtering implementation, displayed remarkable stability for lower PT settings, showing minimal degradation. However, at higher PTs, particularly in the 800 ns and higher settings, we observed an unexpected trend where resolution initially degraded with increasing ICR but then showed improvement at very high count rates. This non-monotonic behavior may relate to the system’s PUR algorithms becoming more selective at longer shaping times, as can be observed in Fig. 5 ▸(*a*). When examining the relationship between peaking time and energy resolution, DANTE+ exhibited optimal performance with longer peaking times at low count rates, though these settings necessarily limit maximum event throughput.

FalconX’s model-based SITORO algorithm demonstrated comparable performance, consistently maintaining below 200 eV resolution even at high count rates. This stability is noteworthy, as it demonstrates the system’s ability to preserve spectral quality at higher counting rates. When paired with the ARDESIA-16 detector, the high-rate filter emerged as the optimal configuration. The system’s adaptive processing algorithm effectively mitigates the typical resolution degradation often observed in high-throughput X-ray spectroscopy detectors.

**Figure 6:**
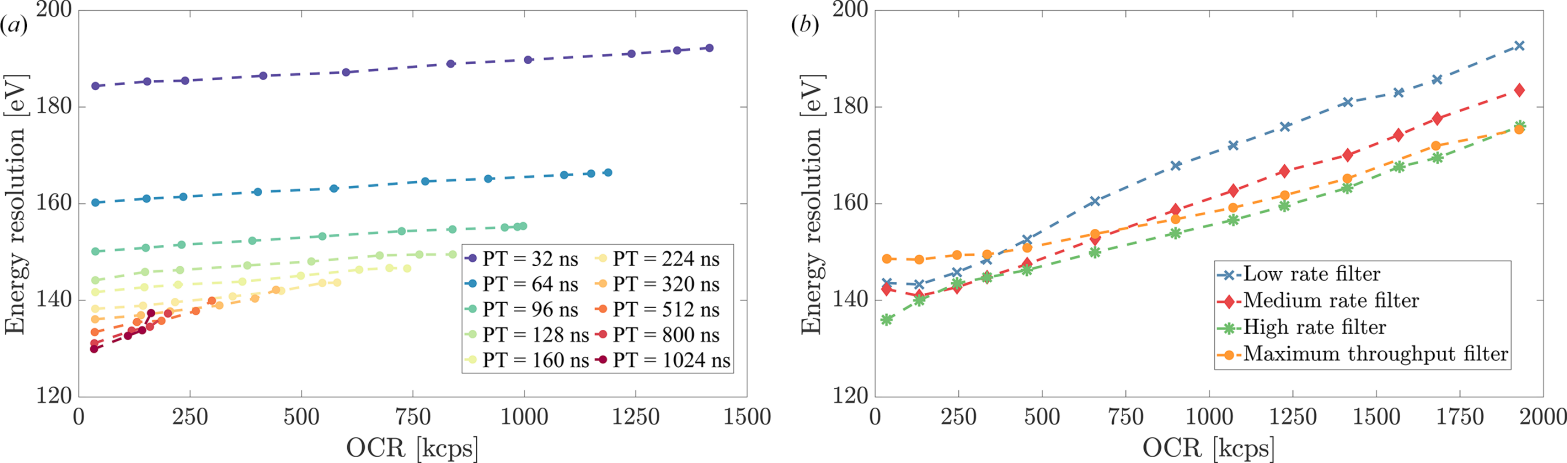
Energy resolution variation with OCR for (*a*) DANTE+ and (*b*) FalconX DPPs. (*a*) Multiple curves demonstrate the impact of different peaking times on resolution and maximum OCR, showing how signal processing parameters influence event throughput and spectral quality. (*b*) Due to the adaptive nature of FalconX’s signal processing, different filter settings produce similar outcomes as OCR increases.

The investigation of energy resolution in relation to OCR provides valuable insight into the performance limitations and signal processing capabilities of the DPPs. Fig. 6 ▸, which shows a comparison between FWHM and OCR, highlights the relationship between system throughput and Mn-*K*_α_ peak resolution for both DANTE+ and FalconX.

Fig. 6 ▸(*a*) illustrates the performance characteristics of DANTE+. Color-coded lines represent different PT configurations, highlighting the trade-off between energy resolution and maximum event throughput. For fixed PT values, the curves demonstrate stable energy resolution with minimal degradation. However, clear differences emerge between PT settings, which require precise tuning to achieve optimal performance. Shorter values of PT show excellent resolution stability over a wide OCR range; for instance, with a 32 ns PT, the resolution degrades only by 8 eV (from 184 eV to 192 eV), while reaching an OCR of 1.4 Mcps. Similarly, with a 96 ns PT we observed a 5 eV degradation (from 150 eV to 155 eV) at a final OCR of 1 Mcps. In contrast, longer peaking times exhibit a more rapid resolution loss with increasing OCR and progressively lose data collection capability once they exceed a specific OCR. The 1024 ns PT setting, for instance, provided the best starting resolution at 130 eV, but could not sustain output count rates beyond 200 kcps. This phenomenon illustrates the delicate balance between energy resolution and DPP settings.

Fig. 6 ▸(*b*) provides a different perspective by showing FalconX system’s model-based signal processing approach, illustrating the effects of the different processing filters. With subtle variations between different filter settings, the system shows a consistent trend of slight but gradual energy resolution degradation as OCR increases. However, the FalconX architecture demonstrates more consistent performance across filter configurations than traditional approaches at significantly higher OCRs. For example, with the HR filter, the resolution degrades by approximately 33 eV (from 143 eV to 176 eV) as the OCR increases from low rate to its maximum of 2 Mcps.

### 3.2. Throughput performance

**Figure 7:**
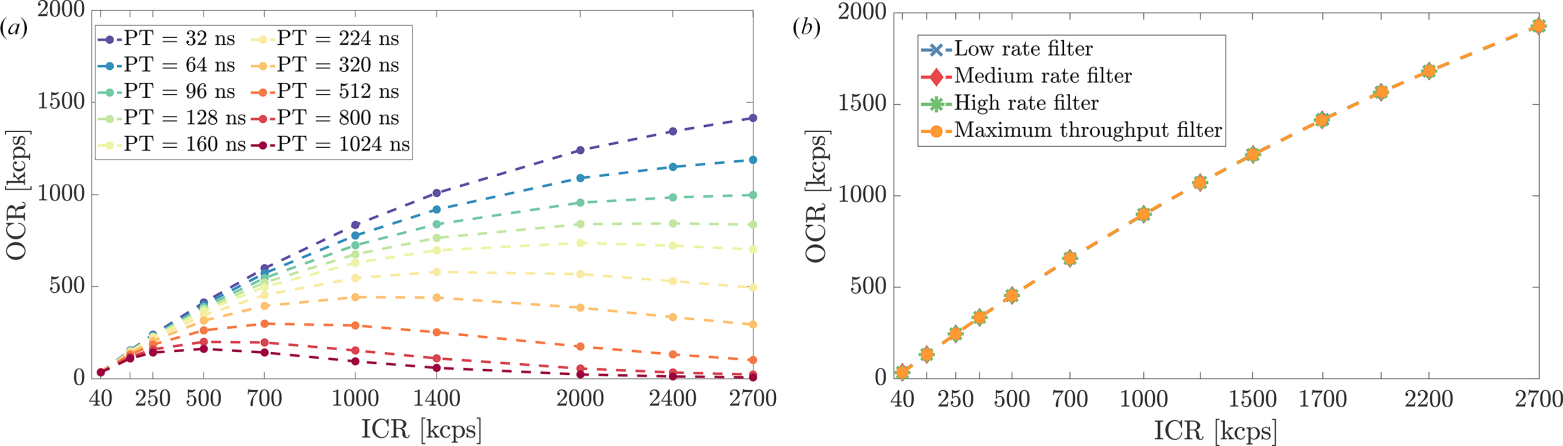
OCR versus ICR performance comparison. (*a*) DANTE+ exhibits different curves with varying PT settings, showing how shorter peaking times allow for higher event throughput but potentially compromise resolution at extreme count rates. (*b*) FalconX exhibits a consistent response across different filter settings.

The ability to process high event rates without significant losses in resolution is crucial in high-count-rate environments such as synchrotron beamlines, where fast data acquisition is essential. Maximum OCR defines the upper limit of detectable events, influencing the system’s capability to handle large volumes of data. OCR was measured by analyzing the total number of events processed per second at varying ICRs, while dead time was calculated as in equation (1) to quantify the systems’ temporal unavailability for event processing,
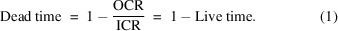
As shown in Fig. 7 ▸, the relationship between OCR and ICR varies significantly between the two systems, reflecting their different processing approaches. At the maximum tested ICR of 2.7 Mcps, FalconX demonstrated higher event throughput, achieving an OCR of 2.0 Mcps, while DANTE+ operated with a maximum OCR of about 1.4 Mcps, using the shortest PT.

**Figure 8:**
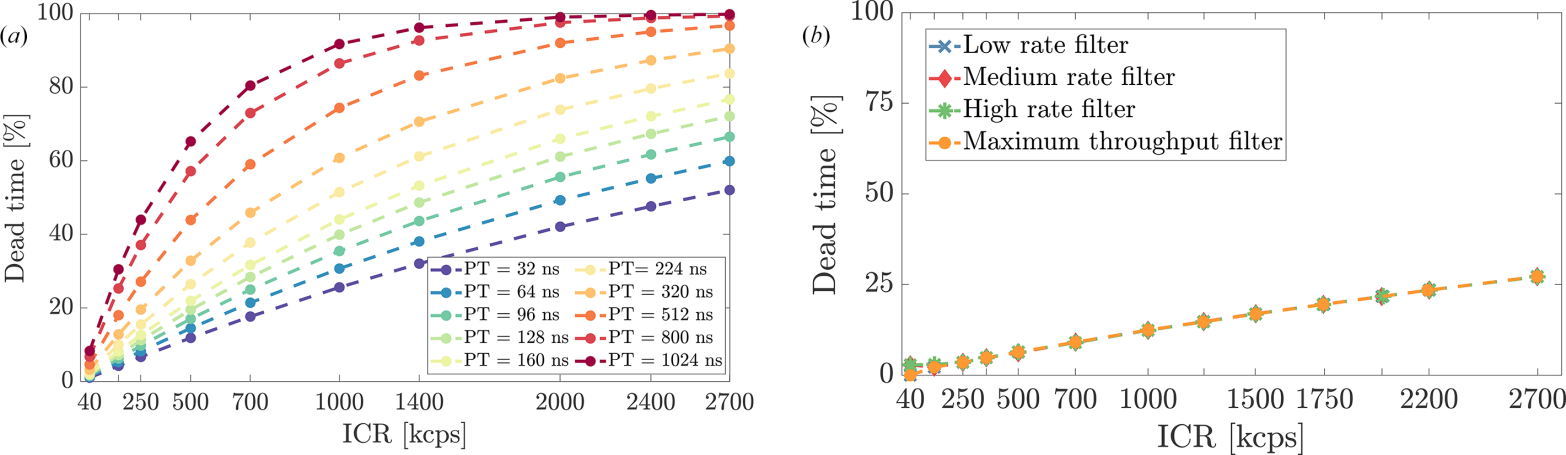
Dead time analysis across ICRs. (*a*) DANTE+ displays dead time behavior that is highly dependent on the PT configuration; optimizing the PT is essential to balance energy resolution and event throughput while minimizing dead time. (*b*) FalconX demonstrates uniform dead time characteristics across different processing modes, though this consistency in event throughput may come at some cost to energy resolution.

Dead time progression with ICR of both systems, visualized in Fig. 8 ▸, provides another view of these performance trade-offs, specifically highlighting the strong dependence of DANTE+’s throughput on the chosen shaping time, in contrast to the more uniform behavior of FalconX across its filter modes: DANTE+ [Fig. 8 ▸(*a*)] showed a dead time increase with ICR highly dependent on trapezoid parameters, with a steeper increase for longer PT values. FalconX [Fig. 8 ▸(*b*)], by comparison, showed minimal variation in event throughput across different filter configurations, and overall lower dead time percentages across a wider range of count rates.

### 3.3. Spectral quality

Spectral analysis is critical for the evaluation of X-ray detection systems, as spectral quality directly impacts peak identification accuracy and quantitative analysis capabilities of synchrotron applications. Therefore, a comprehensive assessment of spectral metrics across operational conditions provides critical information about detector performance in synchrotron environments.

At high count rates, pulse pile-up can significantly degrade spectral quality. Efficient PUR algorithms identify overlapping pulses, ensuring accurate energy determination. Peak position stability across varying count rates is critical not only for maintaining consistent energy calibration but also for preventing measurement artifacts, such as apparent compositional changes in XRF maps.

**Figure 9:**
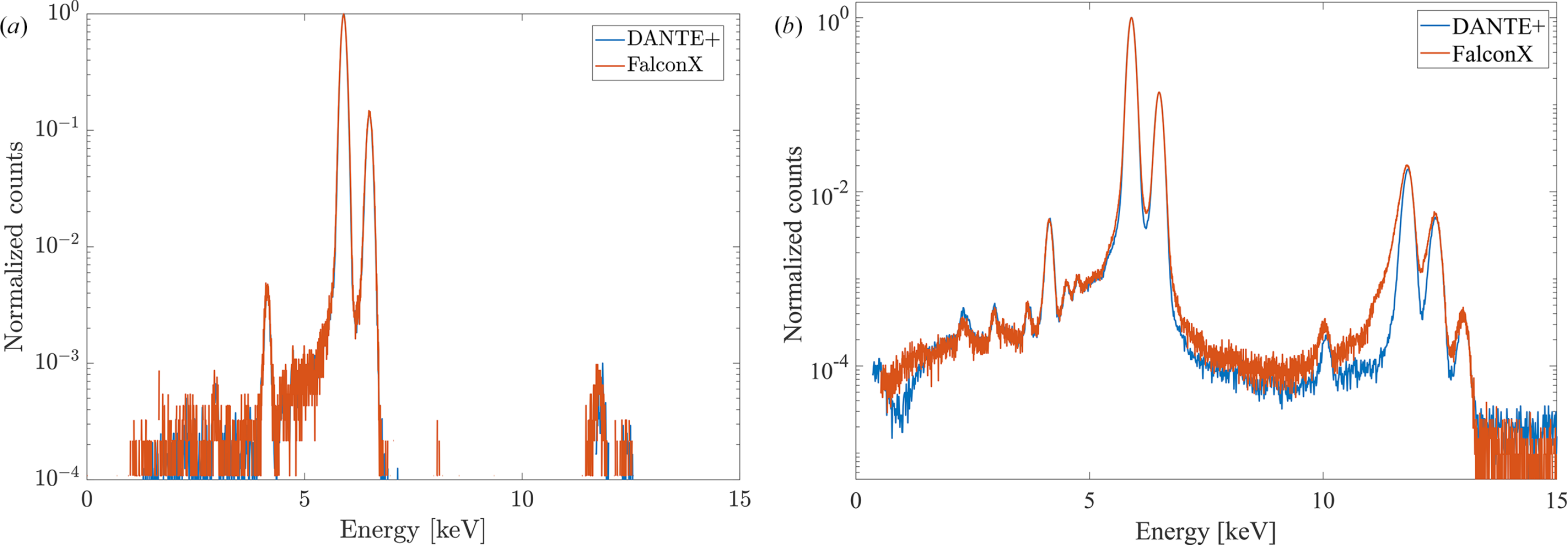
Normalized energy spectra comparison between DANTE+ and FalconX at different input count rates. (*a*) Lower ICR (50 kcps). (*b*) Higher ICR (1 Mcps) showing pile-up characteristics.

Spectral analysis revealed comparable PUR performance between the two systems. Fig. 9 ▸ depicts the normalized energy spectra from both DANTE+ and FalconX at a low ICR of 50 kcps [Fig. 9 ▸(*a*)] and at a higher ICR of 1 Mcps [Fig. 9 ▸(*b*)]. The logarithmic scale highlights subtle differences in background counts and peak characteristics, which may be attributed to different PUR settings of the two systems. With these specific settings, at the lower ICR, the Mn-*K*_α_ peak FWHM was 139 eV for DANTE+ and 141 eV for FalconX. At 1 Mcps ICR, these values degraded to 155 eV for DANTE+ (with the 96 PT setting) and 156 eV for FalconX.

Fig. 9 ▸(*b*) shows spectra collected in high-rate configuration of the experimental setup, where pile-up effects are most apparent. While both systems produced remarkably clean spectra with effective PUR, FalconX exhibited somewhat increased artifacts in the Mn-*K*_α_ pile-up region and in the region where typical double-amplitude pile-up events are present, likely due to sub-optimal configuration of its PUR filters. Although we did our best to optimize PUR settings, we do not exclude that, with additional optimization, these parameters would likely yield improved performance.

**Figure 10:**
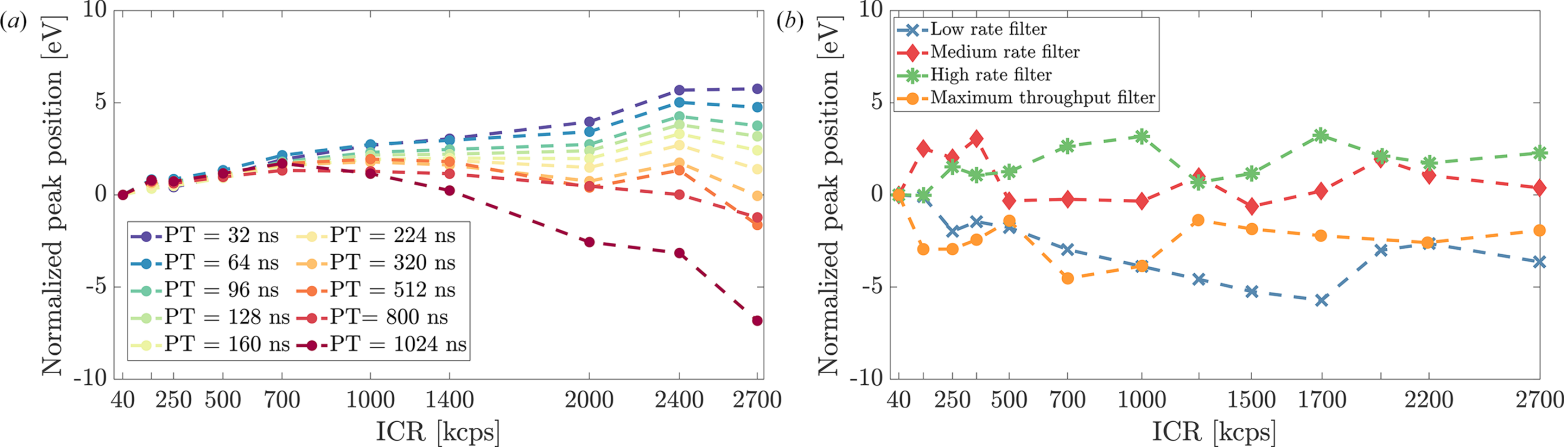
Normalized peak position deviation versus ICR. (*a*) DANTE+ peak stability with varying PT settings. (*b*) FalconX peak stability under different filter configurations.

Peak position stability as a function of input count rate is presented in Fig. 10 ▸, with measurements normalized to peak positions at 40 kcps to isolate count-rate-dependent effects.

Peak stability is a critical factor for energy calibration in spectroscopic applications, and was excellent in both systems. DANTE+ demonstrated particularly uniform peak position stability with medium peaking times (+3 eV deviation across the entire count rate range with 320 ns PT), but slightly less stable at shorter and longer PTs, with a monotonic positive drift for the shortest PT value of +5.3 eV and a negative drift of −6.2 eV for the longest PT setting. FalconX maintained consistent peak positions across its operating range, with the medium throughput configuration exhibiting the best results (+3.8 eV for the medium and high rate filter, −6.7 eV for the maximum throughput filter). This stability ensures reliable energy calibration in XRF mapping experiments where target element concentrations vary.

### 3.4. Discussion

The comparative analysis of the performance of DANTE+ and FalconX with ARDESIA-16 reveals significant insights into the performance trade-offs inherent in high-count-rate spectroscopy applications. Both systems demonstrate robust performance in terms of event throughput and energy resolution; however, they embody distinct design approaches that yield different strengths under varying experimental conditions.

DANTE+ provides the flexibility to fine-tune various operational parameters to optimize performance for specific experimental requirements, although this level of control requires additional manual intervention. In contrast, FalconX adopts an adaptive, plug-and-play design that minimizes user input, offering a more automated, albeit less customizable, solution.

Table 1 ▸ summarizes the key performance metrics for both systems across a range of operating conditions selected based on their relevance to typical experimental use cases.

DANTE+ achieves a superior energy resolution of 125 eV at low ICR compared with FalconX’s 135 eV when both systems are configured for the best possible energy resolution. However, as the ICR increases to 2.7 Mcps, the maximum OCR observed with our setup for DANTE+ is limited to 1.4 Mcps, while FalconX reaches 2.0 Mcps. At these higher rates, the energy resolution degrades to 195 eV for DANTE+ versus 175 eV for FalconX, indicating that FalconX better maintains resolution under extreme photon throughput conditions. Under the operational condition of 1 Mcps ICR, a common experimental condition, DANTE+ exhibits an energy resolution of 155 eV accompanied by a dead time of approximately 30%, whereas FalconX achieves a comparable resolution of 156 eV with a significantly lower dead time of 12%. The systems offer similar OCRs for targeted resolution thresholds (750 kcps for <150 eV and 1.0 versus 1.2 Mcps for <160 eV).

This comparative analysis reveals complementary strengths that may align with different experimental priorities in synchrotron applications. DANTE+ prioritizes optimal energy resolution and particularly resolution stability, achieving this through sophisticated trapezoidal filtering and PUR. This approach proves particularly valuable for applications where precise energy discrimination is critical. However, it involves extensive characterization and parameter tuning and inherently involves trade-offs between resolution and event throughput due to the nature of the trapezoidal filter: longer peaking times improve resolution but increase dead time and reduce maximum count rate capabilities. However, the system effectively balances these factors, maintaining excellent resolution even at moderate to high rates. FalconX’s model-based SITORO approach instead demonstrates advantages in throughput performance while maintaining very good energy resolution under the low-energy conditions evaluated in this work. The system’s adaptive processing enables consistent performance across varying count rates without requiring manual parameter optimization: by modeling the detector response, this approach can extract more information from complex pulses, reducing the need to reject pile-up events and thereby improving overall event throughput. This proves particularly valuable in applications where maximizing data collection efficiency is paramount to reduce the experiment time. Under the tested high-rate conditions at 5.9 keV, the ARDESIA-16 detector systems demonstrated outstanding performance when paired with either DPP technology. Further characterization would be required to evaluate the system’s perfromance with the *K*-lines of higher-*Z* elements, which was beyond the scope of this work.

## 4. Conclusions

Experiments using high-end DPPs can achieve ultra-high count rate requirements. To cope with the quest for ultra-high-rate detectors demanded by next-generations synchrotron beamlines, we conducted an evaluation of the performances of the ARDESIA-16 detector at different input count rates and with two DPPs – XGLab’s DANTE+ and XIA LLC’s FalconX.

The integration of advanced DPPs with the ARDESIA-16 detector system significantly improves event throughput capabilities in synchrotron radiation experiments. These enhancements enable faster, high-resolution measurements. Our comprehensive evaluation of DANTE+ and FalconX reveals distinct performance profiles suited to different experimental requirements. The trapezoidal filtering approach of DANTE+ delivers exceptional energy resolution stability and precision at moderate count rates. Conversely, FalconX’s model-based SITORO algorithm demonstrates excellent event throughput performance. Both systems, paired with the ARDESIA-16 detector, achieve excellent performance metrics, including energy resolution below 160 eV at 1 Mcps ICR and maximum output count rates exceeding 1 Mcps while maintaining resolution below 200 eV. While performance with more complex, multi-line spectra or at higher energies warrants dedicated investigation, these results provide a baseline for system optimization.

Finally, it is worth noting that, while this work focused on the analysis of two systems that embody different processing methodologies, the landscape of high-performance DPPs also includes other prominent systems. A comprehensive analysis could be extended in future works to include other well established systems, such as Xspress 3 (Quantum Detectors Ltd, 2025 ▸) or newly developed DPPs (Abba *et al.*, 2024 ▸), to provide an even broader perspective on the state-of-the-art.

## Figures and Tables

**Table 1:** Comparative performance of DANTE+ and FalconX digital pulse processors

Performance metric	DANTE+	FalconX
Best resolution FWHM (low ICR)	125 eV	135 eV
Highest OCR (PUR implemented, ICR 2.7 Mcps)	1.4 Mcps	2.0 Mcps
Energy resolution at maximum OCR	195 eV	175 eV
Energy resolution at 1 Mcps ICR	155 eV (PT = 96 ns)	156 eV
Dead time at 1 Mcps ICR	30% (PT = 96 ns)	12%
Maximum OCR for energy resolution < 150 eV	750 kcps	750 kcps
Maximum OCR for energy resolution < 160 eV	1 Mcps	1.2 Mcps
